# Supplementary material for: Association between oral microbiome and seven types of cancers in East Asian population: a two-sample Mendelian randomization analysis
Source: Front Mol Biosci. 2023 Nov 21;10:1327893. doi: 10.3389/fmolb.2023.1327893 (PMC10702768; doi:10.3389/fmolb.2023.1327893)
Supplement: Supplementary file 1 [file DataSheet1.ZIP › Supplementary Materials/MR plots for tongue/tongue═╝/Pancreatic cancer/╥╚╧┘░⌐_╔╕╤í╜ß╣√/pheno.236_to_pancreatic cancer_leave_one_out.pdf]

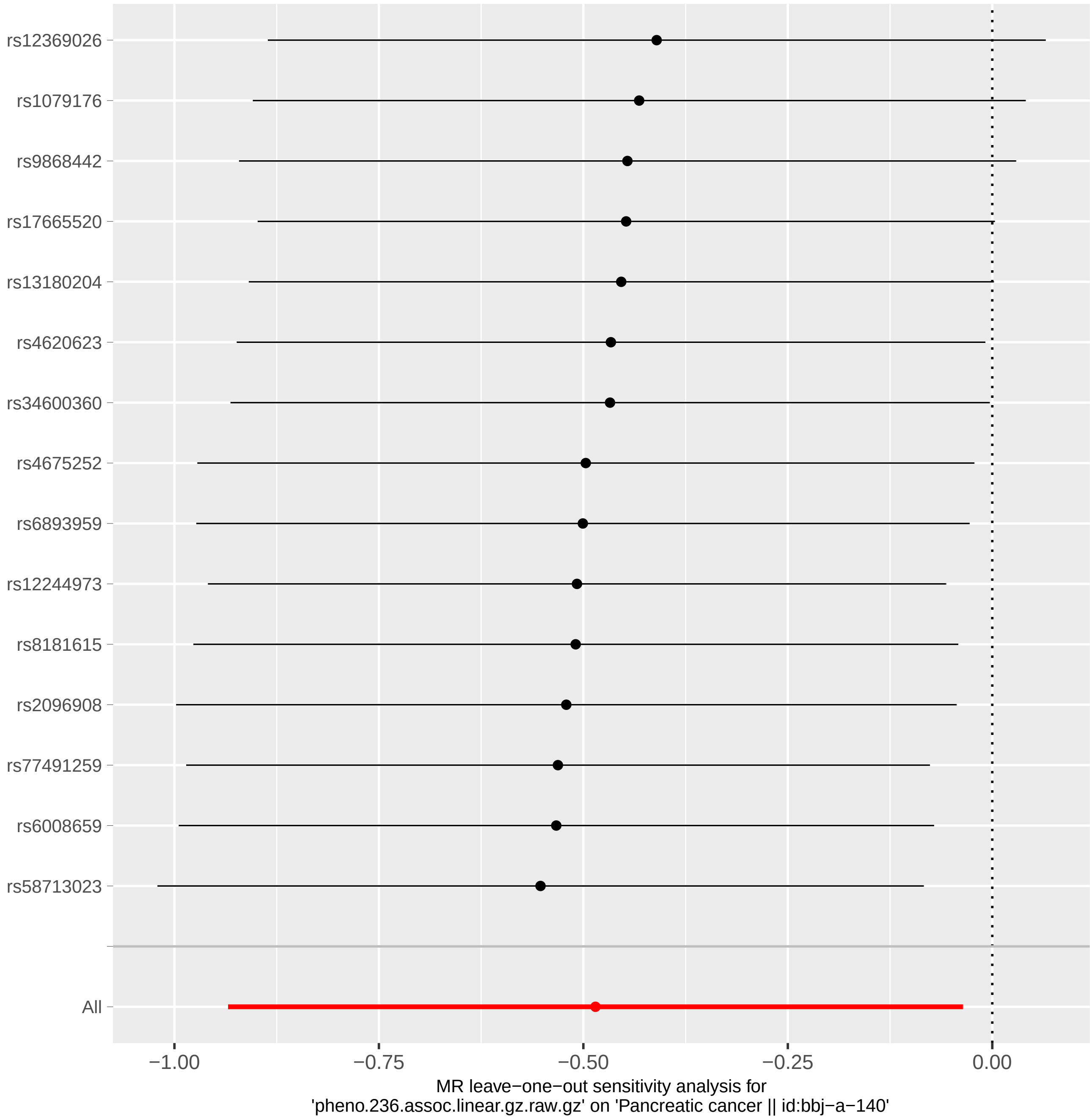

rs12369026
rs1079176
rs9868442
rs17665520
rs13180204
rs4620623
rs34600360
rs4675252
rs6893959
rs12244973
rs8181615
rs2096908
rs77491259
rs6008659
rs58713023
All
−1.00
−0.75
−0.50
−0.25
0.00
MR leave−one−out sensitivity analysis for
'pheno.236.assoc.linear.gz.raw.gz' on 'Pancreatic cancer || id:bbj−a−140'
